# Reconstruction of Scalp and Forehead Defects: Options and Strategies

**DOI:** 10.7759/cureus.41479

**Published:** 2023-07-06

**Authors:** Deepak Krishna, Manal M Khan, Rahul Dubepuria, Gaurav chaturvedi, Ved Prakash Rao Cheruvu

**Affiliations:** 1 Department of Burns and Plastic Surgery, All India Institute of Medical Sciences, Bhopal, Bhopal, IND; 2 Department of Trauma and Emergency, All India Institute of Medical Sciences, Bhopal, Bhopal, IND

**Keywords:** scalp flap, electric burn of scalp, forehead reconstruction, scalp reconstruction, scalp defect

## Abstract

Background

Aesthetic reconstruction of scalp and forehead defects with local flaps and minimal donor site morbidity is the primary goal of coverage. While selecting the coverage technique, essential factors such as size, location, and components of a defect, hair-bearing or non-hair-bearing nature of skin, status of the exposed skull, need for radiation, patient condition, availability of local tissue, and the potential for hairline distortion should be kept in mind.

Materials and methods

This is a retrospective analysis in which 54 patients who underwent soft tissue reconstruction of the scalp and forehead defects were included. The defect size was categorized into four groups: small: <4 cm^2^, medium: 4-50 cm^2^, large: 50-200 cm^2^, and very large: >200 cm^2^. Reconstruction of all defects was done according to the defect's size, location, and depth.

All patients were regularly followed at intervals of two weeks, six weeks, and three months, respectively. The outcome was evaluated in terms of flap survival, flap coverage scale, the requirement of secondary treatment, postoperative complications, and final aesthetic appearance.

Results

In 54 consecutively treated patients with scalp and forehead defects, the male-to-female ratio was 2:1, and the overall mean age of participants was 34.8 years, ranging from 0.5 to 66 years. The most common etiology of the defect was trauma (16; 29.6%), and the most common location of the defect was combined (16; 29.6%). Rotation flap and primary closure were the most commonly performed procedure, each 12 (22.2%) in number. Out of 12 primary closure cases, two patients developed wound dehiscence because of infection. All cases of skin grafting healed well. All cases of transposition flap with skin grafting at the donor site went uneventful, and the dog ear at the base was revised later. One case of the bipedicle flap in which partial graft loss occurred at the donor area was managed with regrafting.

Two cases of single rotation flap, one double rotation flap, and one free latissimus dorsi muscle flap developed distal necrosis. The excellent aesthetic outcome was found in all cases of primary closure and single and double rotation flaps.

Conclusions

Local flaps have an architecture similar to the recipient site, and low donor site morbidity results in an aesthetically more pleasant outcome. In our experience, scalp defects up to 50 cm^2^ were covered with the local scalp flaps with primary closure of the donor area. Defects ranging from 50 to 200 cm^2^ required local scalp flap with skin grafting at the donor area. Free tissue transfers are usually needed when the defect is very large, devoid of the periosteum, or with the calvarial defect.

## Introduction

The scalp and forehead are specialized areas of the skin composed of multiple soft tissue layers that cover the skull. Physically, the scalp protects the cranial vault from trauma and external foreign materials and is also aesthetically crucial because of the presence of hairs. The forehead is a nonhairy, aesthetically significant area containing the frontalis muscle, the frontal branch of the facial nerve. Different causes of exposure to the cranium, a subcutaneous bone, are avulsion injury, electric burn, post-tumor resection, radiation, and infection [1-5]. The scalp also is a common site for skin cancer because it is directly exposed to sunlight [6,7]. The presence of the loose areolar connective tissue provides the ability to elevate scalp flaps in the subgaleal plane as well as allow separation between upper layers and pericranium in traumatic injury. Scalp avulsion injuries mainly occur in working places and are commonly seen in females due to their comparatively long hair [8]. Exposed scalp and forehead wounds can lead to osteomyelitis of pericranium and severe neurological problems such as meningitis and brain abscess as the infection from the scalp wound can go to the cranial cavity by valveless emissary veins [9].

The reconstructive goals are to achieve coverage with well-vascularized soft tissue, which is cosmetically acceptable with minimal donor site morbidity [6,10]. Reconstructive options for scalp and forehead defects include primary closure, secondary healing, skin grafting, local and regional flaps, or free tissue transfer [6]. While selecting the coverage technique, essential factors such as size, location, defect components, need for radiation, hairy or nonhairy nature of skin, surrounding tissue condition, and the potential for hairline distortion should be considered [4,10,11]. Knowledge of neurovascular structures and mobility in different regions is essential in planning coverage options. The general condition of the patients, previous treatment history, state of exposed bone, and requirement of adjuvant therapy postoperatively also determine the choice and stages of the coverage technique. Long-standing exposure of the skull can lead to necrosis of the skull's outer part, which must be removed before coverage. Because of the robust blood supply of the scalp, local flaps can be utilized in the coverage of small to large scalp defects with a good cosmetic outcome. There is variation in the mentioned coverage scale of different scalp flaps in the literature [4,7,10,11]. So, in this study, we analyzed the condition of the exposed cranium, coverage scale, and outcome of different flaps required for coverage of scalp and forehead complex soft tissue defects.

## Materials and methods

In this study, a retrospective analysis was performed using the medical records of 54 patients who had undergone soft tissue reconstruction of the scalp and forehead defects in the Department of Burns and Plastic Surgery at All India Institute of Medical Sciences, Bhopal, from January 2016 to December 2021. This study was approved by the Institutional Research Review Board (Ethics code: IHEC-LOP/2021/IM0347), and informed consent was obtained from all participants before enrolment.

**Figure 1:**
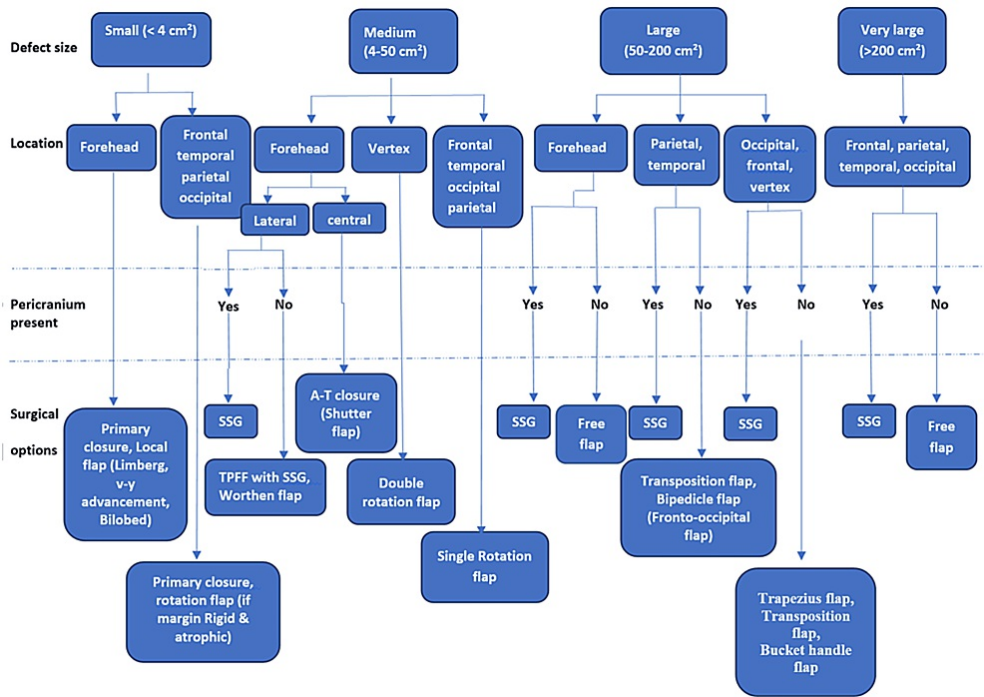
Algorithm based on size, location, and depth of scalp and forehead defect SSG: Split skin grafting; TPFF: Temporoparietal fascial flap.

Patient data, including age, sex, concomitant medical illness, etiology, defect size, location and depth of the defect, history of previous treatment, and type of surgery, were recorded. The defect size was categorized into four groups: small: <4 cm^2^, medium: 4-50 cm^2^, large: 50-200 cm^2^, and very large: >200 cm^2^. The location was considered as a peripheral region (forehead, frontal, temporal, or occipital), central region (parietal or vertex), or combined when involved in more than one area [12]. Defects were reconstructed based on the defect size, location, and status of the pericranium (Figure 1).

Whenever possible, small-sized defects at the forehead and scalp were managed by primary closure or local flap with primary closure of the donor area. Medium-sized defects were closed by a single rotation flap in the peripheral area and a double rotation flap in the central area. Large- and very large-sized defects, where the donor site could not be closed primarily, were covered by skin grafting when the pericranium was intact and transposition (unipedicle or bipedicle) or free flap when the pericranium was lost.

All surgeries were performed under general anesthesia after freshening the margin, milling the outer surface of bone if exposed for a long duration, and taking adequate margins in case of tumor resection. Skin grafting was done when the wound bed appeared vascular. The arc of the rotation flap was marked at the periphery four to five times the defect margin to be approximated to ensure primary closure of the donor area. After injecting an adrenaline solution of 1:200,000 dilution at the marking site, an incision was made, and the flap was elevated in the avascular plane with preservation of the pericranium. The closure was tension-free, and back cut or galea scoring was performed when required.

The transposition flap was marked equal to defect size with a length-to-width ratio of up to 1:1 to 1:3 with the named vessel at the pedicle base.When the length-to-width ratio increased more than 1:3, or the flap distal margin crossed the midline, a bipedicle flap was planned to increase the vascularity of the flap. The dog ear at the flap base in the transposition flap is not excised immediately because it would narrow the flap pedicle and decrease its blood supply. The donor site of the flap was covered with skin grafting, and pressure dressing was applied. Sutures were removed 14 days postoperatively.

Free Latissimus dorsi muscle flap with skin grafting over it was used to cover a very large scalp defect. Muscle was harvested in a lateral position under general anesthesia, and anastomosis was done with the superficial temporal artery and vein in the same position. The loose dressing was done over a skin graft to avoid pressure at the anastomosis site. A free radial forearm flap was used for the coverage of bone-deep large forehead defect. Before the opening of microclamps after anastomosis, 5,000 units of unfractionated heparin were administered; later, 60 mg of low-molecular-weight heparin (LMWH) was given twice daily for five days.

All patients' regular follow-ups were noted at intervals of two weeks, six weeks, and three months, respectively. The outcome was evaluated in terms of flap survival, flap coverage scale, the requirement of secondary treatment, donor site morbidity, postoperative complications, and final aesthetic appearance. A subjective assessment of the final aesthetic outcome was done by another plastic surgeon as poor, good, and excellent.

## Results

Fifty-four consecutively treated patients with scalp and forehead defects were included in this retrospective study. The male-to-female ratio was 2:1, and the overall mean age of participants was 34.8 years, ranging from 0.5 to 66 years. The distribution of gender, age, comorbidities, etiology, and location of defect are summarized in Table 1. The most common etiology of the defect was trauma (16; 29.6%), and the most common site of the defect was combined (16; 29.6%).

**Table 1:** Demographic characteristics of participants CAD: Coronary artery disease.

Patients’ characteristics	Overall (N = 54)
*Gender and age*	
Male	36 (66.6%)
Female	18 (33.3%)
Male:female ratio	2:1
Mean age and range	34.8 (0.5-66)
*Comorbidities*	
Diabetes	4 (7.4%)
Hypertension	6 (11.1%)
Asthma	2 (3.7%)
CAD	2 (3.7%)
Smoking	10 (18.5%)
*Etiology of defect*	
Trauma	16 (29.6%)
Avulsion of scalp	8 (14.8%)
Electric contact burn	12 (22.2%)
Tumor resection	8 (14.8%)
Post-infective	6 (11.1%)
Nonhealing ulcer	4 (7.4%)
*Location of the defect*	
Forehead	10 (18.5%)
Frontal	4 (7.4%)
Parietal	6 (11.1%)
Temporal	2 (3.7%)
Occipital	6 (11.1%)
Vertex	10 (18.5%)
Combined	16 (29.6%)

Comparative analysis of different surgical procedures utilized for reconstruction of forehead and scalp defects in terms of average defect size, number of procedures, complications, aesthetic outcome, and operative time are presented in Table 2. Single rotation flap and primary closure of defect were the most commonly performed procedures, each 12 (22.2%) in number.

**Table 2:** Outcome analysis of different techniques

		Primary closures, V-Y, Limberg	Single rotation flaps	Double rotation flaps	Transposition flaps	Bipedicle flaps	Distant flaps	Free skin grafts	Free flaps
Number of cases (n)		12	12	6	8	2	2	10	2
Mean size of the defect in cm^2^		3.8	33	32.3	75.7	188	65	80.4	220
Complications (n)		Dehiscence (2)	Tip necrosis (2)	Tip necrosis (1)	0	Partial graft loss (1)	0	0	Distal necrosis (1)
Secondary treatment (n)		Secondary suturing (1), rotation flap (1)	Secondary healing (1), re-advancement of the flap (1)	Drilling of bone followed by skin grafting	0	Regrafting (1)	0	0	Drilling of bone followed by skin grafting
Mean operative time in minutes		18	92	112	76	82	155	38	234
Donor site morbidity		−	−	−	+	+	++	+	+++
Aesthetic outcome (n)	Excellent	12	12	6	0	0	0	0	0
	Good	0	0	0	2	1	1	6	0
	Poor	0	0	0	6	1	1	4	2
Defect size (n)	Small	12	2	0	0	0	0	0	0
	Medium	0	10	6	0	0	1	2	0
	Large	0	0	0	8	2	1	6	0
	Very large	0	0	0	0	0	0	2	2
Defect depth (n)	Superficial	8	4	0	0	0	0	8	0
	Pericranium	4	8	6	7	1	2	2	2
	Bone	0	0	0	10	0	0	0	0
	Dura	0	0	0	0	1	0	0	0
Location of the defect (n)	Forehead	4	2	1	0	0	1	2	0
	Frontal	2	1	0	0	0	0	1	0
	Parietal	2	2	1	0	0	0	1	0
	Temporal	0	2	0	0	0	0	0	0
	Occipital	0	3	0	1	0	1	1	0
	Vertex	4	2	2	0	0	0	2	0
	Combined	0	0	2	7	2	0	3	2

Out of 12 cases of primary closure, two cases developed wound dehiscence because of infection, out of which one patient managed with secondary suturing and the other required a rotation flap. All cases of primary skin grafting healed well. All cases of transposition flap with skin grafting at the donor site went uneventful, and the dog ear at the base was revised later.

Out of 12 cases of single rotation flap, two patients developed distal tip necrosis, one had a narrow pedicle base and managed with secondary healing, and the other had a short arc of rotation and managed with re-advancement of the flap. One case of the bipedicle flap in which partial graft loss occurred at the donor area was handled with regrafting. In one case of double rotation flap who developed distal tip necrosis and in one case of free latissimus dorsi muscle flap with distal 2-cm necrosis of muscle, exposed skull bone was managed by drilling of bone by round burr up to diploic layer to allow granulation tissue to appear and was followed by coverage with skin grafting.

The excellent aesthetic outcome was found in all cases of primary closure, single and double rotation flap. The requirement of milling of exposed bone or removal of necrotic bone was compared in post-electric contact burn and other patients as shown in Table 3.

**Table 3:** Management of exposed cranium in electric contact and other etiology scalp defects

Groups	Milling of the outer layer of the cranium required (n)	The average timing of coverage after injury, in days (range)	Primary reason
Post-electric burn (n = 12)	8 (66%)	60 (16-180)	Removal of necrotic bone before flap coverage as a primary procedure in all cases
Others (n = 42)	4 (9.5%)	14 (2-21)	To promote granulation followed by skin grafting (in two cases as a primary procedure and in the other two cases as a secondary procedure)

## Discussion

Cosmetic reconstruction of scalp defects requires hair restoration by local scalp tissue and preservation of standard hair patterns and lines [1,10]. Local scalp flap skin is similar to lost skin in terms of color, hair growth, and thickness, which gives an esthetically more pleasant outcome [1,13]. When choosing reconstructive options, consideration of size, location of scalp and forehead defects, patient condition, and goals allows coverage with the best outcome [4,10,11,14,15]. Here, the authors present an algorithm approach to reconstruct scalp and forehead defects based on size, location, and components while considering the cosmetic factor when selecting different options.

Primary closure of defect after undermining of margin of edges or rotation advancement flap provides the best aesthetic outcome. The primary closure of wounds even less than 3 cm in diameter requires wide undermining because of the limited mobility of the scalp [16]. Galea scoring perpendicular to the line of maximum tension and parallel to the sub-aponeurotic blood vessels facilitates the mobility of the flap and reduces pressure at the closure site [17]. Healing by secondary intention can be an effective method but is not considered by most reconstructive surgeons as it causes hairless scars. Thus, this option can be considered for the hairless and bald regions [16].

Small defects of less than 4 cm^2^ were closed primarily. At the forehead region, primary closure needs elliptical excision of extra tissue, which lengthens the final scar and can lead to distortion of anatomical landmarks, while local flaps like Limberg, V-Y advancement, single or bilateral linear advancement, and bilobed flap provide closure without secondary deformity. Small defects at the peripheral scalp region are usually closed primarily as skin in these regions is lax. Still, rotation flaps were performed in the central area where the skin is less mobile or when the surrounding skin is scarred and rigid.

**Figure 2:**
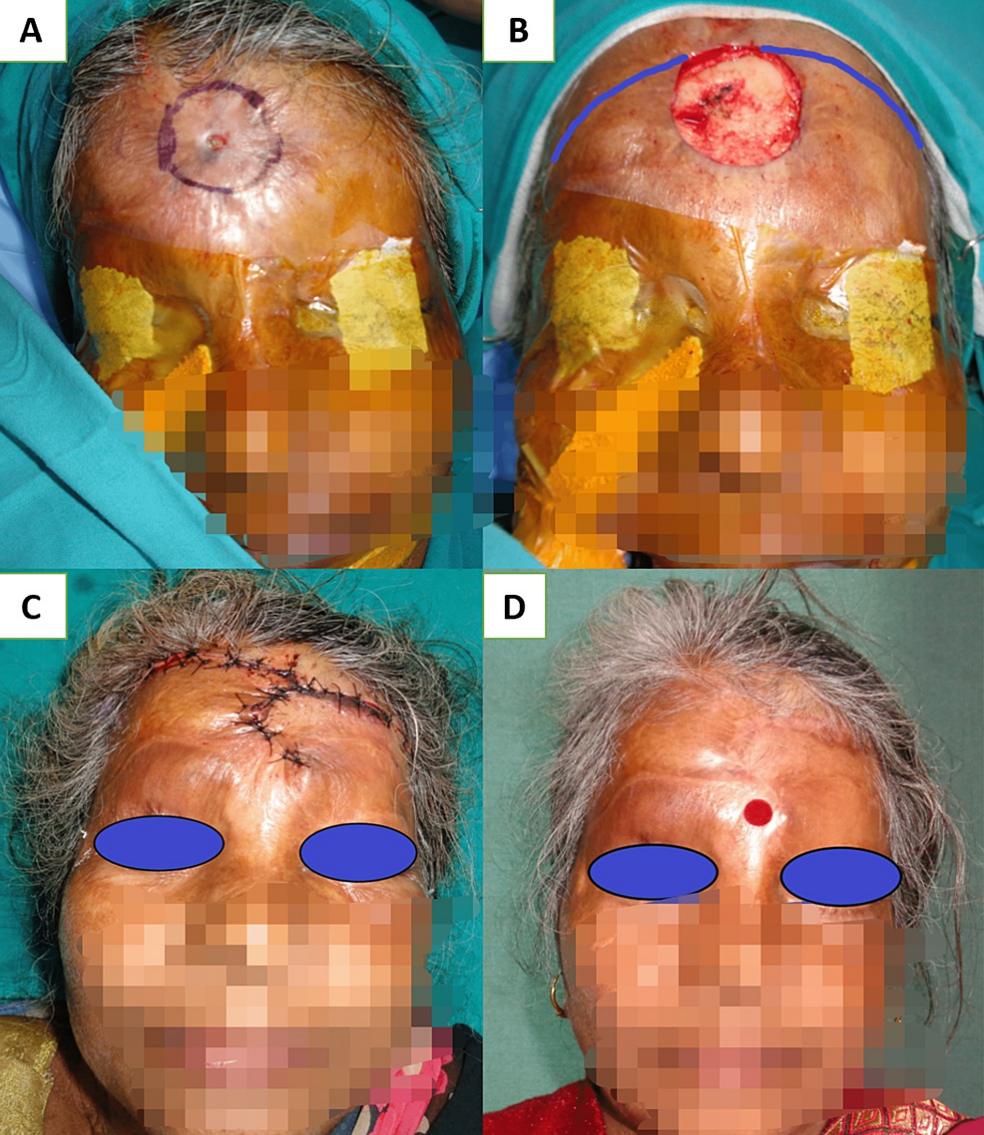
(A) Nonhealing ulcer of size 1 x 1 cm with surrounding scarring in the mid-forehead region; (B) 5 x 5 cm size defect after excision and marking of double rotation advancement flap; (C) one-week follow-up; and (D) three months follow-up

**Figure 3:**
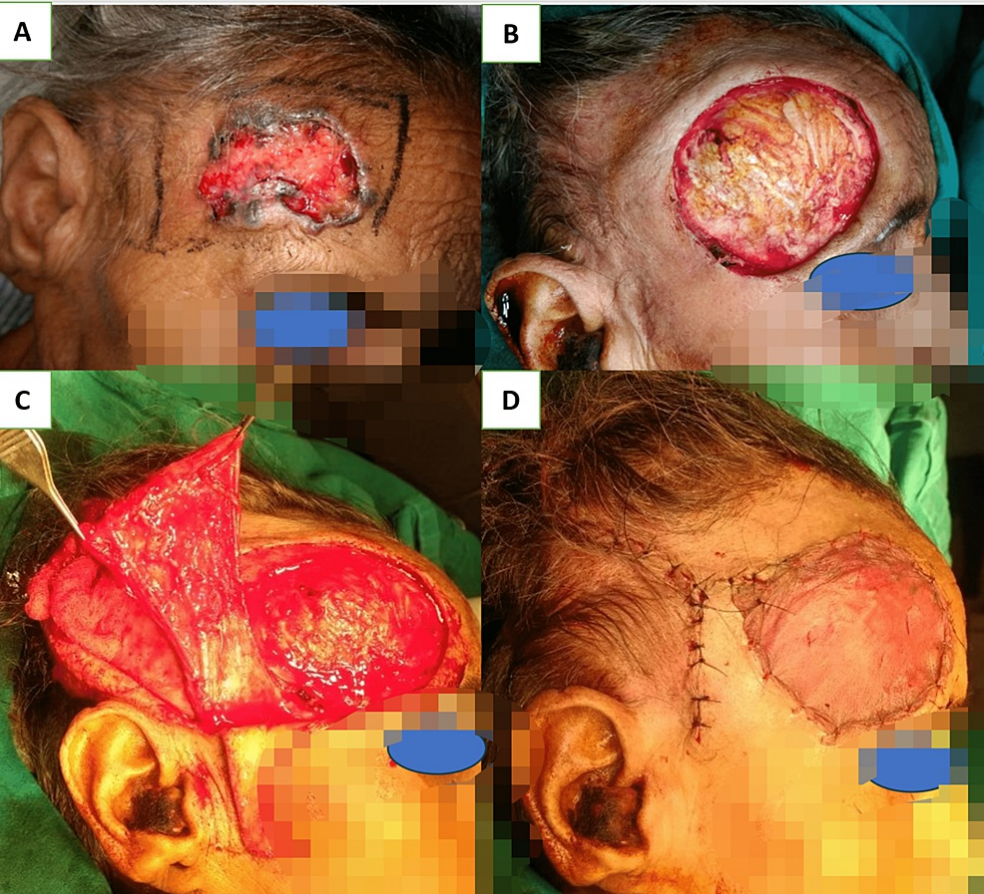
(A) BCC right-side forehead; (B) 7 x 6 cm size defect after excision; (C) temporoparietal fascial flap elevated of size 10 x 6 cm; (D) wound was covered with a TPF flap with SSG over it, and the donor site was closed primarily. BCC: Basal cell carcinoma; TPF: Temporoparietal fascial; SSG: Split skin grafting.

**Figure 4:**
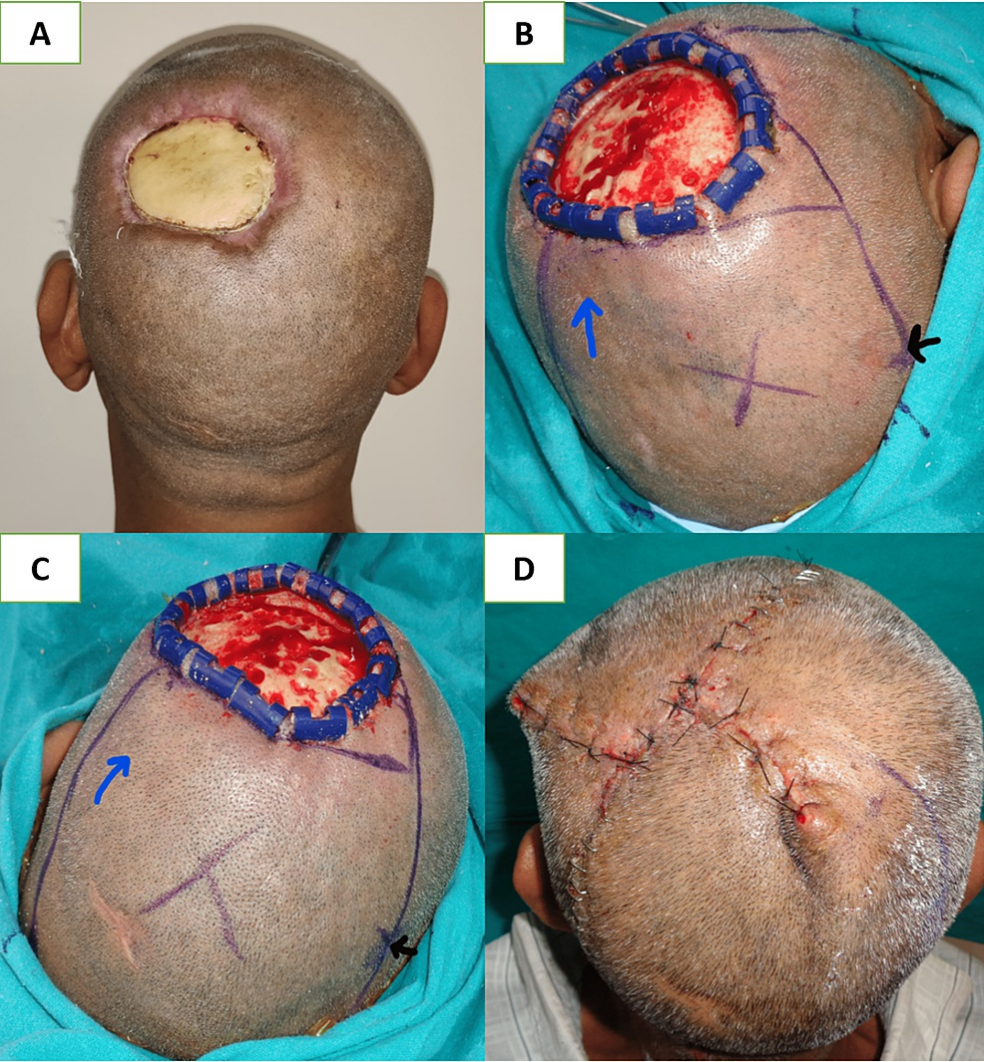
(A) Post-electric burn defect of 6 x 8 cm size in the left parietal region; (B) and (C) marking of double opposing rotation flap, X and Y (blue arrow denotes the direction of rotation, and the black arrow denotes the center of the arc), along with debridement of the necrotic outer layer of bone by round burr; and (D) one-week postoperative result

**Figure 5:**
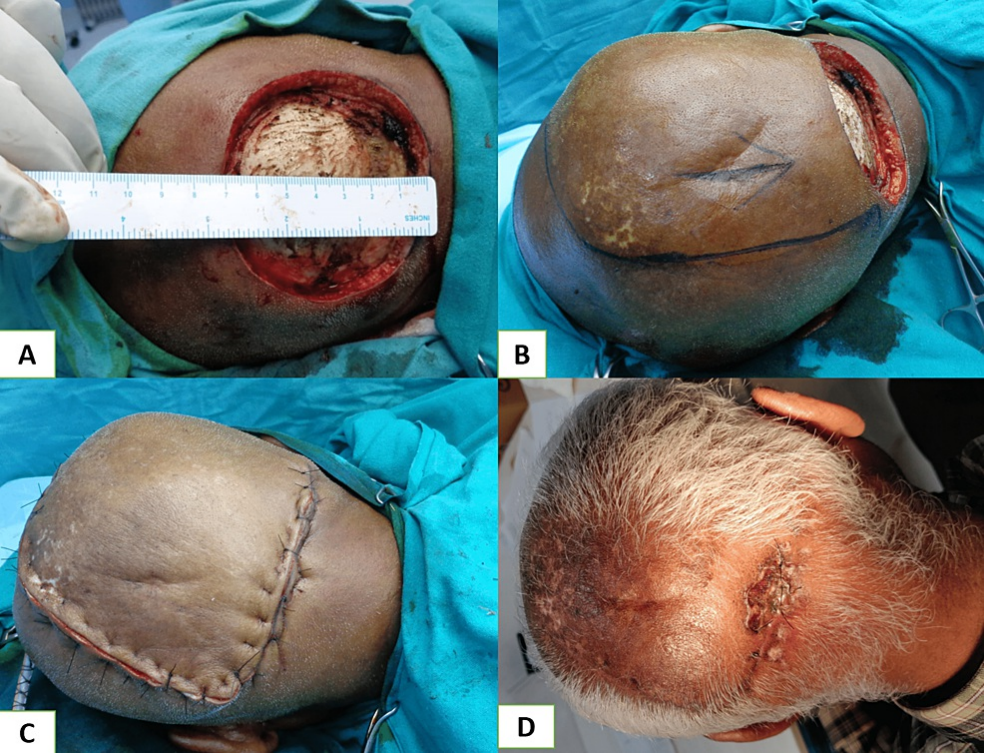
(A) Post-SCC excision defect of size 7 X 7 cm at the occipital region; (B) marking of rotation flap; (C) defect closure intraoperatively; and (D) one-month postoperative result SCC: Squamous cell carcinoma.

Medium-sized defects at the central forehead region were managed with an A-T flap with a transverse incision along the hairline when defects were located at the middle and upper parts of the forehead (Figure 2, Panels A-D). On the other hand, when defects were found at the lower part of the forehead, transverse incisions were made just above the eyebrow to avoid approximation of the medial end of the eyebrows. Lateral forehead defects can be managed with a skin graft alone over an intact pericranium or with a temporoparietal fascial flap with an overlying skin graft (Figure 3, Panels A-D), and the rotation flap can be worthened if the pericranium is lost [18]. Defects up to 50 cm^2^ at the vertex region were closed with a double opposing rotation flap (Figure 4, Panels A-D). They closed at the peripheral region with a single rotation flap without hairline distortion (Figure 5, Panels A-D). Primary donor area closure in the rotation flap resulted in an excellent aesthetic outcome.

**Figure 6:**
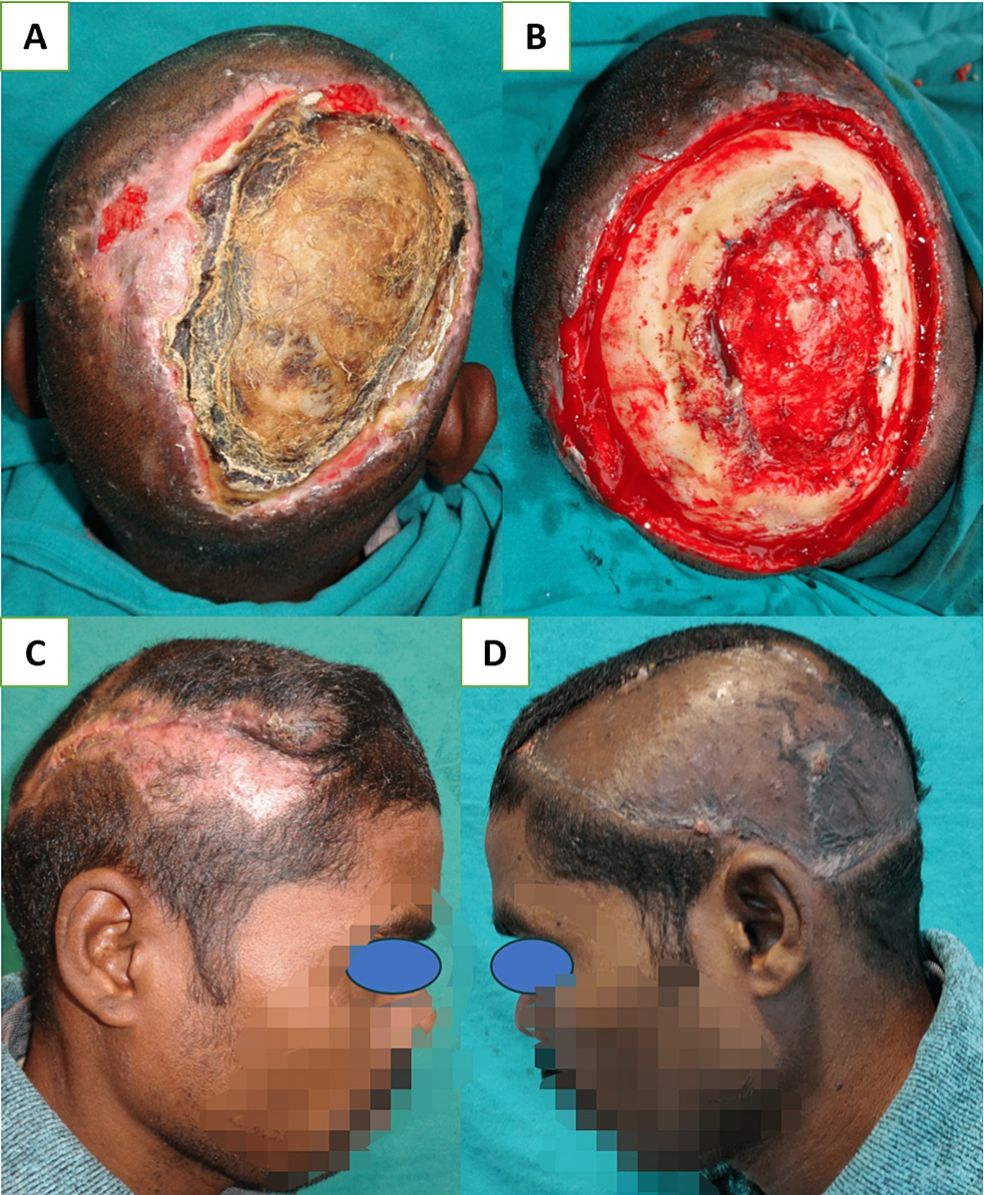
(A) Post-electric burn defect of 16 x 12 cm size in the right parietal region; (B) debridement of bone shows the involvement of the outer table in the central part and layer of granulation tissue formed between the two layers of the cranium; (C) the defect is covered with a left anteriorly based transposition flap; and (D) donor site is covered with skin grafting

**Figure 7:**
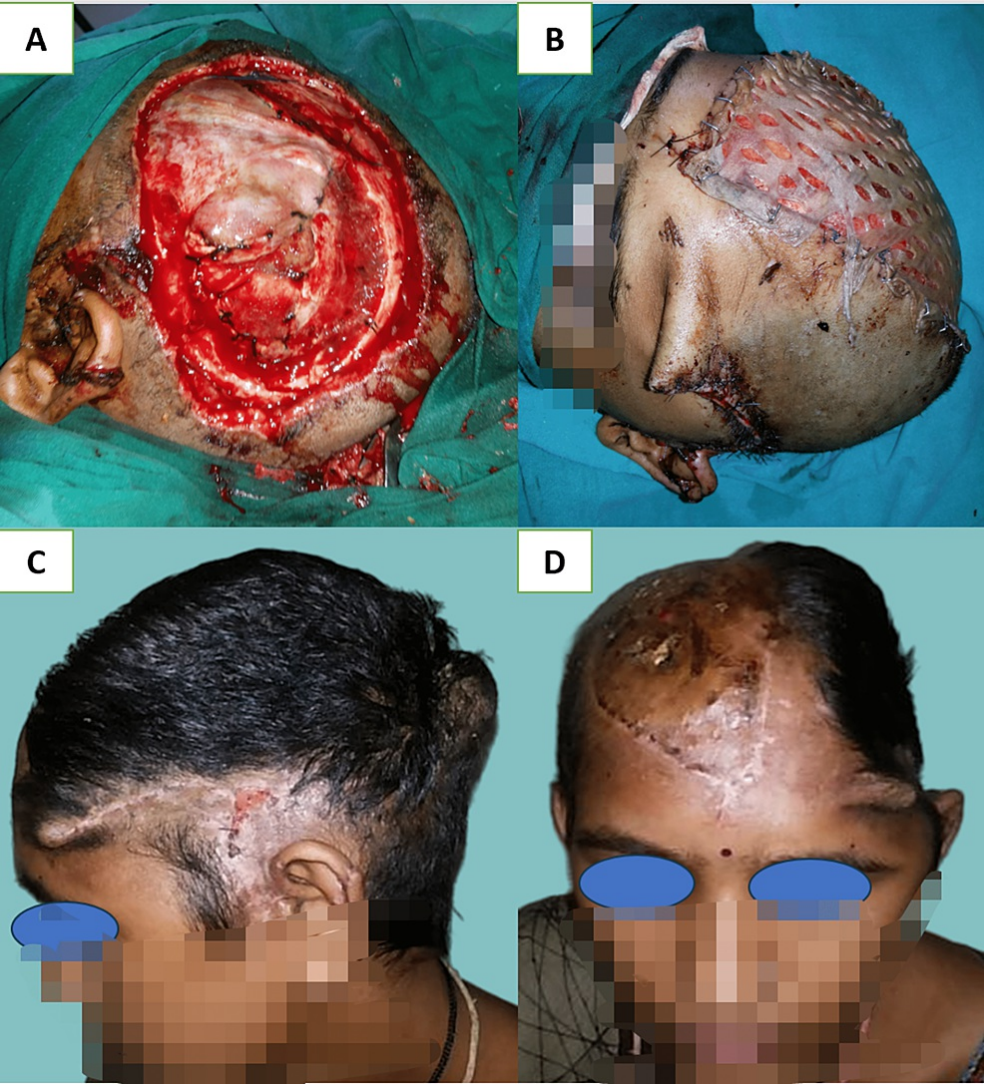
(A) Scalp avulsion injury left the temporoparietal region, defect size of 18 x 10 cm, with bone loss and exposed dura mater; (B) the defect is covered with a bipedicle fronto-occipital flap with SSG at the donor site; (C) and (D) lateral and front views at three months follow-up SSG: Split skin grafting.

Large-sized scalp defects, between 50 and 200 cm^2^, were managed by a single (Figure 6, Panels A-D) or bipedicle transposition flap (Figure 7, Panels A-D) from a less cosmetically appealing area (occipital and temporal) to a more cosmetically appealing area like frontal, parietal, and vertex. The donor site of transposition flaps should be kept in a less aesthetic place so that nearby long hairs can cover the skin-grafted area [17]. While designing these local flaps, attention should be given to including at least one major blood vessel at the base of the flap. Defects in less cosmetical areas like occipital and temporal, which can be hidden by long hairs, were managed with a skin graft. The temporal area has deep temporal fascia and temporal muscle, which can be covered with a skin graft. Bone-deep defects at the temporal site usually occur following oncological resection or in avulsion injury, which requires free flap coverage. In avulsion injuries, you may find an injured vascular pedicle sometimes, which necessitates using a vein graft or arterio-venous loop to use recipient vessels outside the trauma zone [3].

**Figure 8:**
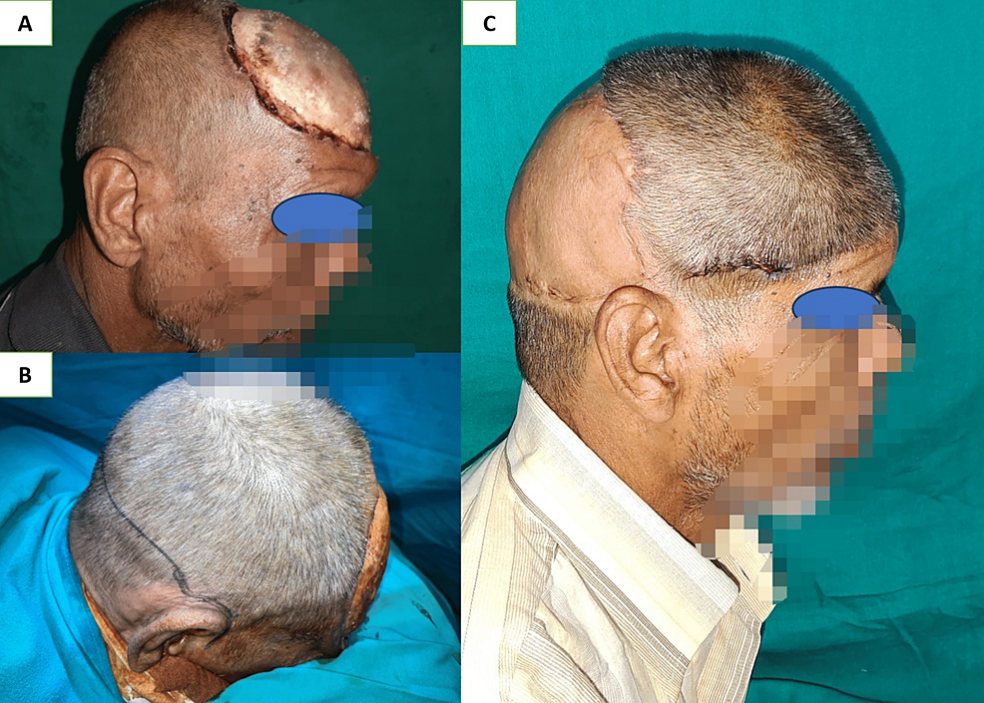
(A) 14 x 14 cm sized post-electric burn defect in the frontoparietal region; (B) bucket handle flap marking, flap incision which is 28 cm equal to the distance between both side roots of the helix at the level of posterior margin of the defect; and (C) postoperative results at three months

Fronto-occipital (Figure 7, Panels A-D) and temporo-temporal bipedicle flaps (Figure 8, Panels A-C) have excellent coverage scales for bone-deep temporal and frontal defects associated with injury to vascular pedicle or where free flap could not be performed due to any reason [19,20]. Large full-thickness defects at the occipital region can also be covered with a pedicle trapezius flap. But these distant pedicle flaps like trapezius, pectoralis, and latissimus dorsi myo-cutaneous flaps have the disadvantage of being nonhairy and bulky with donor site morbidity when used for scalp defects [21], and there are chances of flap ischemia in the distant flap when used in comorbid patients [22].

In complication, distal tip necrosis of the local flap may occur due to a narrow pedicle or excessive tension at the wound margin resulting from the inadequate arc of rotation. The burrow’s triangle at the base should not be excised at the same sitting to maintain the flap's blood supply. The common causes of graft loss at the donor site are injury to the pericranium either due to improper plane of dissection or scarring at the donor site. These skin-grafted donor sites in the transposition flap can be replaced later on by a tissue-expanded flap for aesthetic improvement [13,23-25]. When postoperative radiation therapy is required in such sized defects, free flaps are more reliable than local flaps because skin graft at the local flap donor site may not tolerate postoperative irradiation [5].

Coverage with a thick skin graft was performed in large forehead defects when the pericranium was intact, giving acceptable aesthetic results. Skin graft at the forehead region is commonly performed when a transverse forehead flap is utilized for post-oncological full-thickness cheek defect coverage. Forehead reconstruction with a fasciocutaneous free flap is usually required in post-electric or post-oncological defects [26].

**Figure 9:**
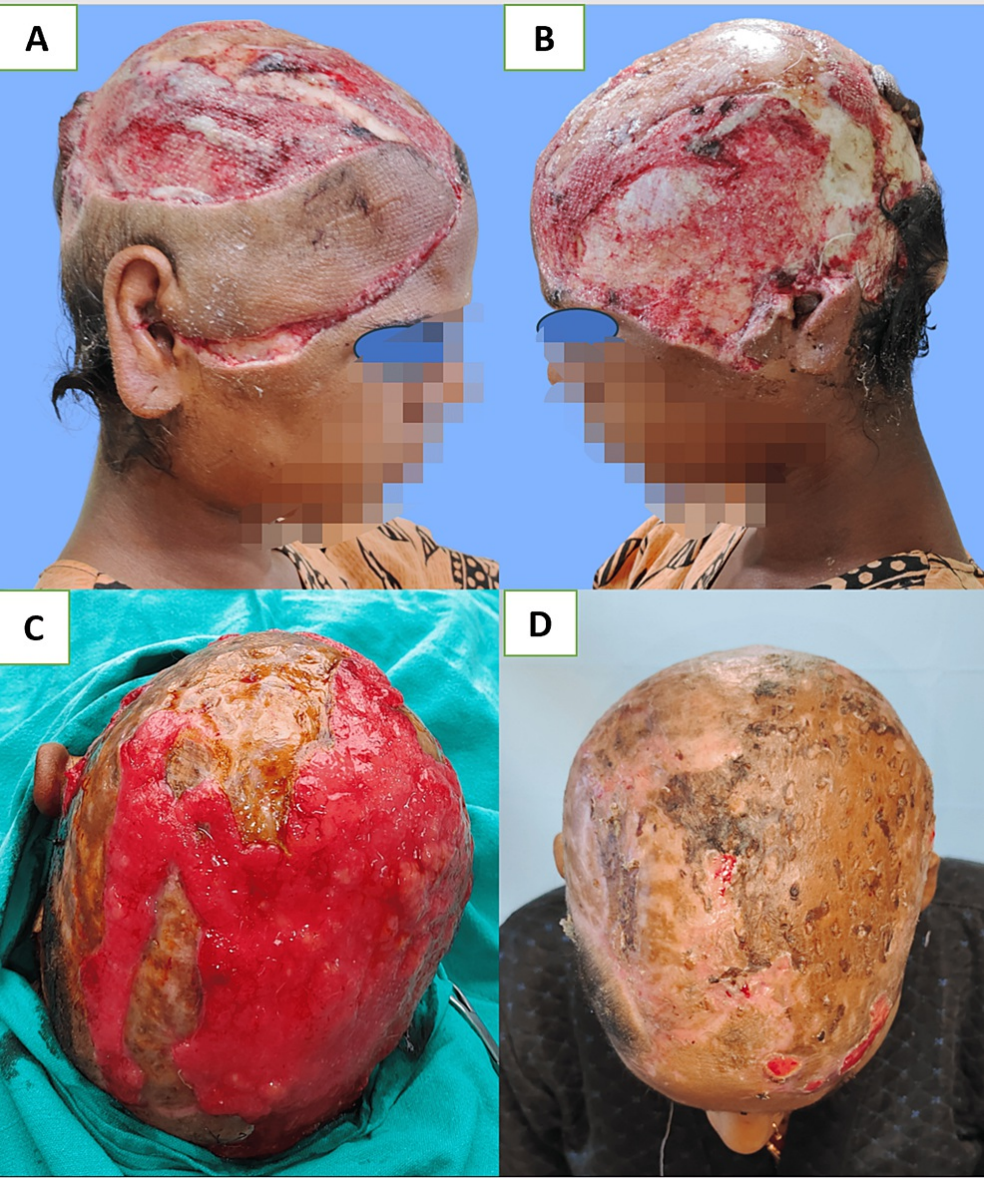
(A) and (B) Right and left lateral views showing near-total avulsion of the scalp; (C) appearance of granulation tissue after milling of the exposed cranium and taken-up skin graft over intact pericranium; and (D) taken-up skin graft after two months

A free split-thickness skin graft can be tried after drilling down to the diploic layer of bone to improve the granulation process for coverage of broad defects larger than 100 cm^2^ even if there is loss of pericranium and other options are not feasible. This technique can be used in patients who cannot tolerate prolonged anesthesia or those with associated cardiac comorbidity [24]. We used this technique of drilling the skull up to the diploic layer in cases where bone got exposed after necrosis of the distal part of the flap and in patients with near-total scalp defects that are not fit for free tissue transfer (Figure 9, Panels A-D). Another described technique is immediate skin grafting after milling the outer table, which can fasten the healing in scalp defects devoid of pericranium due to post-oncological resection or trauma [27]. Delayed healing, graft loss, and later, the trophic ulcer may occur when a skin graft is applied at the damaged pericranium or bleeding inner table after removal of the outer table [26,27]. The dermal regenerative template has also been used after removing the external table to improve the cosmetic outcome and durability of the graft [28]. Koenen et al. showed excellent dermal regenerative template application results after partial removal of the outer table of the skull in elderly patients with aggressive scalp malignancies [29]. However, the increased cost of treatment and risk of infection is also described. Vacuum-assisted closure (VAC) devices can improve wound beds when vital structures are exposed along with a dermal regenerative template [30]. To promote secondary healing, we applied VAC with multiple discharging wounds at the scalp in one case.

**Figure 10:**
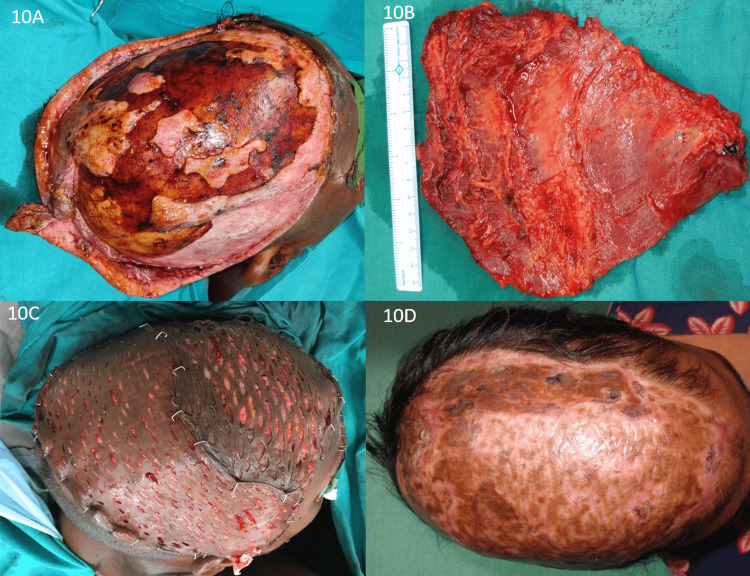
Scalp avulsion by Chara cutting machine: (A) Scalp avulsion injury in the right temporoparietal and left parietal regions, defect size of 20 x 15 cm; (B) harvested free latissimus dorsi muscle flap; (C) wound covered as well as LD muscle flap and SSG over the muscle; and (D) three months follow-up

Local tissues may be inadequate in nearly total or very large scalp defects, and tissue expansion or free tissue transfer may be the only alternatives [4,10,12,31,32]. Scalp defects > 200 cm^2^ were covered with a free latissimus dorsi muscle flap with an overlying skin graft when pericranium was absent (Figure 10, Panels A-D). Free tissue transfers are mainly required after post-tumor resection with loss of pericranium or calvarial defect with exposed dura. Latissimus dorsi muscle flap, anterolateral thigh flap, para scapular flap, rectus abdominal flap, radial forearm flap, etc. are some commonly used free flaps [33-35]. Among the free flaps, muscle flaps with an overlying skin graft are less bulky than fasciocutaneous flaps, which later provide better contour. Some studies suggest hair transplantation can be performed on the skin paddle of the fasciocutaneous flap after thinning the flap to improve aesthetic outcomes [36]. Latissimus dorsi is a muscle-free flap with a broader scale of coverage, which limits its use due to a change of position intraoperatively and donor site morbidity [33,34]. Fasciocutaneous free flaps are preferred when the calvarial bone is reconstructed using implants as there are no chances of atrophy, scarring, and better tolerability to radiation [37]. Also, when secondary cranioplasty is planned, fasciocutaneous flaps are preferred over muscle flaps. Furthermore, the tissue of the microvascular free flap is often too bulky, and the nonhair-bearing reconstruction is esthetically unpleasant but more durable than a skin graft.

We encountered only a few cases of skull bone loss and scalp defect and have no experience with bony reconstruction. When reconstructing full-thickness skull defects in the acute stage, the size and location of bone defects and expected intracranial pressure are essential determinants. Different indications of cranioplasty are protection from trauma, cosmesis, and the putative “syndrome of trephined” [38]. According to van Driel et al., small- to medium-size bone defects (≤5-7 cm) can be left without replacement if not located at the forehead or occipital region, which are cosmetic and pressure-sensitive areas, respectively [35]. They also recommended morselized bone graft for small-size defects and calvarial or rib graft for medium-size defects. Large-size bony defects require a vascularized rib graft with a free latissimus dorsi muscle flap cover [39]. It is advisable not to use nonvascularized bone grafts and prosthetic material when postoperative radiotherapy is planned. Bone resorption and infection are the most common complication following cranioplasty leading to revision surgery [40]. When concomitant dura defects are not amenable to primary closure, they can be closed with artificial patches or a nonvascularized fascia graft.

In post-electric scalp defects, debridement of the exposed skull is required in 66% of patients, which is significantly higher than that in other patients (9.5%). The mean timing of coverage of scalp defects is 60 days in electric contact scalp defects compared to other patients, which are 14 days (Table 3). The reason behind bone involvement in electric contact scalp defects is the increased depth of injury and delayed presentation to the higher center after healing** **surrounding and other body areas' burned skin. Many times, other associated burn injuries over limbs, chest, abdomen, and genitalia get priority over scalp injury. In some patients, due to long-term exposure to the skull, the necrotic outer layer gets separated from the inner layer in the central part of the defect, and a layer of granulation tissue is formed between two layers.

The local flap was the most commonly used procedure as shown in other studies (Table 4) [7,13,23,41]. The most common cause of scalp defect was accidental (66.6%), e.g., trauma, electric, and avulsion injuries, while malignancy was the most common cause in other compared studies [7,13,21,23]. In our study, the primary reasons for cranial bone debridement were to promote granulation followed by skin grafting in very large scalp defects where other options were not available as a primary procedure before flap coverage in electric contact scalp burn. In other studies, bone involvement was higher and correlated with tumor etiology. Postoperative complications were comparable with other studies and managed with secondary procedures. We have proposed the extended use of local flaps based on their coverage scale compared to other studies [7,21,42].

**Table 4:** Surgical outcome after scalp reconstruction compared with previous studies. NM: Not mentioned; LF: Local flap; FF: Free flap; SG: Skin grafting; DF: Distant flap, PC: Primary closure; ICH: Intracranial hemorrhage.

	Lesavoy et al., 1993 [23]	Nagasao et al., 2011 [41]	Denewer et al., 2011[21]	Cherubino et al., 2013 [7]	Zayakova et al., 2013 [13]	Zhou et al., 2020 [42]	Present study
Reported cases	10	20	42	86	13	173	54
Algorithm based on defect size (cm^2^)	NM	NM	<50, 50-100, >100	<5, 5-20, >20	NM	<4, 4-30, 30-90, >90	<4, 4-50, 50-200, >200
Procedure used	LF	LF, FF	SG, LF, DF, FF	LF, SG, FF	LF	PC, LF, SG, DF, FF	PC, LF, SG, DF, FF
Immediate complications	None	Minor (3)	Major (12), minor (13)	NM	Minor (4)	Minor (8), complex (5)	Minor (7)
Bone involvement	90%	100%	14%	NM	25%	2.8%	14%
Most common etiology	Skin tumor (60%)	ICH (50%)	Skin tumor (100%)	Skin tumor (85%)	Skin tumor (38%)	Skin tumor (100%)	Trauma (66.6%)
Most common procedure	LF (100%)	FF (75%)	DF (57%)	LF (37.5%)	LF (100%)	SG (43%)	LF (55%)

Limitations of the study

Statistical significance between different surgical procedures could not be evaluated because of the wide variety of techniques used for different types of defects, and each group has a small number of cases.

## Conclusions

Most scalp defects can be managed with local scalp flaps with or without skin grafting at the donor site. Compared to local flaps, free tissue transfer is a time-consuming procedure with higher donor site morbidity, which is unsuitable for patients with comorbidity. The selection of a single or double rotation flap depends on the location of the defect rather than the size. Single or bipedicle transposition scalp flaps have a wider coverage scale, are less time-consuming, and are advisable in high-risk patients. Debridement of the outer surface of the cranium is mostly required in delayed presented cases of electric contact burn over the scalp region. Reconstruction with local flap with primary closure of donor site or grafted donor site placed in the less cosmetic area resulted in a better aesthetic outcome.
